# miR-926-3p influences myocardial injury in septic mice through regulation of mTOR signaling pathway by targeting TSC1

**DOI:** 10.18632/aging.204716

**Published:** 2023-05-11

**Authors:** Feng Yan, Qian Wang, Huiyu Yang, Hui Lv, Weiwei Qin

**Affiliations:** 1 Department of Cardiology, The Second Hospital of Shanxi Medical University, Shanxi 030001, People’s Republic of China

**Keywords:** miR-926-3p, TSC1, mTOR signaling pathway, sepsis, myocardial injury

## Abstract

Background: The purpose of this study is to investigate the influence of miR-926-3p on myocardial injury and its mechanisms.

Methods: An animal model of sepsis was constructed by CLP, and animals were randomly divided into 4 groups: C group, miR-926-3p inhibitor group, CLP + NC group, and CLP + miR-926-3p inhibitor group.

Results: Compared with those in C group, echocardiographic parameters remarkably declined in CLP + NC group. Compared with CLP + NC group, miR-926-3p inhibitor group indicated elevated echocardiographic parameters in mice, pathological improvement tendency of myocardial tissues and distinct reduction in cardiomyocyte apoptosis. It could be observed by electron microscopy that the number of lysosomes in miR-926-3p inhibitor group was greatly increased relative to CLP + NC group. Immunofluorescence exhibited that the number of green fluorescent puncta was significantly higher in miR-926-3p inhibitor group as compared to that in CLP + NC group. The autophagic flow was verified by observing the relative expression of LC3II at different times. The results of Western blotting manifested that miR-926-3p inhibitor up-regulated mTOR-related protein expressions and down-regulated the protein expression of p-mTOR. LPS was adopted to induce cardiomyocyte injury *in vitro*, and the results confirmed that, identical to *in vivo* experiments, miR-926-3p inhibitor was able to up-regulate the protein expressions of mTOR-related protein and down-regulate p-mTOR protein expression in cardiomyocytes. After addition of MHY1485, The expression of mTOR-related proteins changes in each group.

Conclusion: Inhibition of miR-926-3p enhances autophagy through regulation of the mTOR signaling pathway, thus ameliorating myocardial injury in septic mice.

## INTRODUCTION

Sepsis as a common critical disease in clinic [1] is a systemic inflammatory response syndrome attributed to excessive secretion of endotoxin lipopolysaccharide (LPS) resulting from microbial infection in blood, while the absence of effective treatments may cause multiple organ failure until death from septic shock [2, 3]. In recent years, more attention has been paid to the harmful effect of endotoxin on the heart. Myocardial injury and cardiac dysfunction, as reported, are already present in early stage of sepsis and markedly increase mortality [4, 5]. Considering that the fatality rate of sepsis-induced myocardial injury is high and there is a lack of safe and effective treatment drugs [6], investigating the pathogenesis of myocardial injury induced by sepsis and searching for effective prevention and treatment approaches are of great importance.

Autophagy has recently been proven to play a new role in embryogenesis and development as well as cell death and immunity, and there exist close relations between autophagy dysfunction and various diseases [7]. Autophagy has dual effects on numerous diseases and contributes to the regulation of metabolism and inflammation [8]. Under normal physiological conditions or following mild attacks, autophagy as an adaptive response can facilitate cell survival. However, insufficient or excessive autophagy resulting from long-term or severe attacks may cause massive degradation of cytoplasmic components and toxin accumulation, either of which is considered a maladaptive response, resulting in cell death [8, 9]. Both *in vivo* model of cecum ligation and puncture (CLP)-induced sepsis in mice and *in vitro* model of LPS-induce cardiomyocyte injury have demonstrated the protective effect of stimulating autophagy on the myocardium, elucidating that autophagy is an adaptive response that can protect cardiomyocytes. From an opposite perspective, directly reducing autophagy with autophagy inhibitors or indirectly reducing autophagy by antioxidants can improve myocardial contractility in mice with LPS-induced sepsis, which supports the conclusion of maladaptive autophagy [10]. Previous studies have indicated that sepsis can trigger autophagy in multiple organs (including the heart) [11, 12], while the increase in sepsis-induced autophagy is a lysosome-dependent process of removing damaged proteins and organelles [13, 14]. Therefore, autophagy modulation offers a novel option for treatment of sepsis [15].

Micro ribonucleic acids (miRNAs), a class of highly conserved endogenous non-coding RNAs, widely exist in eukaryotic cells and participate in regulating multiple biological processes, such as cell growth, development, differentiation and apoptosis [16]. Based on bioinformatics analysis, this study revealed the regulatory effect of miR-926-3p on sepsis-induced myocardial injury, and the influence of miR-926-3p on myocardial injury in septic mice was analyzed, which provides an experimental reference for sepsis diagnosis and the investigation of its effect on cardiac function.

## MATERIALS AND METHODS

### Bioinformatics analysis

The sepsis-related gene expression datasets GSE79962 and GSE12624 were retrieved and downloaded from the Gene Expression Omnibus (GEO) database (https://www.ncbi.nlm.nih.gov/gds/). Then the dataset GSE101639 containing miRNA sequencing data from septic patients was downloaded. RNA-seq data were subjected to quantile normalization with R language limma software package, followed by differentially expressed gene (DEG) analysis (|logFC|>1, *p* < 0.05), principal component analysis (PCA) and cluster analysis. R language software package ggplot2 was used to construct the volcano plot of visually grouped DEGs in the datasets GSE79962 and GSE12624, while the cluster heatmap of DEGs was drawn via R language software package pheatmap. Then in terms of visually grouped DEGs in the dataset GSE101639, the volcano plot and cluster heatmap were drawn using the above-mentioned methods.

### Functional enrichment analysis

Gene ontology (GO) and Kyoto Encyclopedia of Genes and Genomes (KEGG) enrichment analyses were performed for DEGs from the intersection between GSE79962 and GSE12624 datasets. Next, the online database tool DAVID (https://david.ncifcrf.gov/) was utilized for DEG analysis from the level of biological processes to integrate GO terms and construct a biological process network of DEGs. Then the GO pathway diagram and the enrichment analysis diagram of the KEGG pathway of DEGs were plotted using ggplot2 package within the R language environment.

### Gene set enrichment analysis (GSEA)

GSEA (http://www.gsea-msigdb.org/) was carried out for all genes and the GSEA pathway diagram was plotted.

### miRNA target gene prediction

TargetScan, miRDB and miRanda were adopted to predict miRNA candidate target genes. The Venn diagram was drawn using VennDiagram package to predict target genes from candidate genes along with GSE101639. Then the miRNA binding sites in messenger RNA (mRNA) were drawn according to the gene prediction results.

### Experimental animals and model preparation

Forty-eight 3-month-old male C57/BL mice of specific pathogen-free (SFP) grade were purchased from Skbex Biotechnology and housed with comfortable temperature at about 20°C, relative humidity of 50–60% and a 12/12 h light/dark cycle. Then the mice were fed adaptively for one week with routine diet and distilled water. After fasting for 12 h prior to operation, they were anesthetized by intraperitoneal injection of 2% pentobarbital sodium, and then a mouse model of sepsis was constructed by CLP. After operation, subcutaneous injection of normal saline (1 mL) on the back was given to all mice to supplement blood volume and they were fed a normal diet. In this experiment, the animals were divided into four groups: control group (C group), miR-926-3p inhibitor group, CLP + negative control (NC) group, and CLP + miR-926-3p inhibitor group.

### Isolation and incubation of cardiomyocytes

The heart was collected from each neonatal mouse and placed in a pre-cooled phosphate-buffered solution (PBS) culture dish. After separation, the tissue was digested with a proper amount of trypsin solution in a serum bottle at 4ºC overnight. The termination solution composed of 10% fetal bovine serum and 90% Dulbecco’s Modified Eagle Medium (DMEM) was added the next day for reaction in an incubator at 37ºC for 5 min, and the supernatant was discarded. The tissue was digested repeatedly, and the cell suspension was harvested after each digestion and centrifuged at 1,000 rpm for 5 min. Then the supernatant was discarded. Subsequently, the cell suspension was added with 10 mL of culture solution containing 10% fetal bovine serum, 90% DMEM, 100 μL/mL penicillin, 100 μL/mL streptomycin and 0.1 mmol/L BrdU, inoculated in a 100-mm culture dish and incubated in a 5% CO_2_ incubator at 37ºC. Then 1.5 mg/kg LPS was adopted to induce cardiomyocyte injury *in vitro*, and the cardiomyocytes were divided into NC group, miR-926-3p inhibitor group, NC + MHY1485 group, and miR-926-3p inhibitor NC + MHY1485 group [addition of a mammalian target of rapamycin (mTOR) agonist MHY1485 in the medium).

### Measurement of cardiac function

Following the completion of experiment, the mice were anesthetized with isoflurane and fixed on a constant-temperature detection plate for echocardiography using VisualSonics Vevo 770 system with a 30 MHz ultrasonic probe. The following echocardiographic parameters were measured: left ventricular end-diastolic anterior wall thickness (LVAW; d), left ventricular end-systolic anterior wall thickness (LVAW; s), left ventricular end-diastolic posterior wall thickness (LVPW; d), left ventricular end-systolic posterior wall thickness (LVPW; s), left ventricular internal diameter at end-diastole (LVID; d), left ventricular internal diameter at end-systole (LVID; s), and left ventricular ejection fraction (EF).

### Transmission electron microscopy observation

The heart was collected from each neonatal mouse. After rinsing with PBS, the tissue was placed in an ice box and aspirated using a disposable dropper, followed by fixation with 2.5% glutaraldehyde solution and rinsing again with 0.1 mol/L·PBS. The tissue was then immobilized with 1% osmium acid at 4°C for 3 h. After rinsing with 0.1 mol/L PBS, it was subjected to stepwise dehydration with gradient ethanol (30% ethanol for 15 min, 50% ethanol for 15 min, 70% ethanol overnight, 80% ethanol for 15 min, 90% ethanol and 90% acetone mixture (1:1) for 15 min) at 4°C and 100% acetone for 15 min at room temperature, a total of 3 times. Subsequently, the tissue was soaked in pure acetone + embedding solution (1:1) for 2 h at room temperature, and in pure embedding solution at 37°C for 3 h. The heart tissue was transferred to an embedding plate containing embedding solution in an oven at 37°C for 12 h and then in an oven with pure embedding solution at 60°C for 48 h. Semi-thin sections with a thickness of 1 μm were made and stained with Azure blue, which were then observed under light microscopy to determine the myocardial structure of 10 layers. Ultrathin sections were made at a thickness of 80 nm, and they were transferred onto the supporting film copper mesh. The sections were stained with lead citrate for 15 min and rinsed with distilled water 3 times, followed by natural drying and observation via transmission electron microscopy.

### Hematoxylin-eosin (HE) staining

Mice were killed by decapitation, and hearts were immediately dissected and fixed in 4% paraformaldehyde solution. After the tissues were routinely dehydrated and embedded in paraffin, the pathological sections were prepared, which were then stained as per the instructions of HE kits, dried in an oven at 55–60ºC for 20 min, mounted and observed under a fluorescent microscope.

### Detection of GFP-LC3 puncta formation via fluorescence microscopy

Cardiomyocytes transfected with appropriate plasmids were inoculated into 12-well plates at a density of 60% and cultured for 48 h. Cells were fixed with 4% paraformaldehyde in PBS and permeabilized with 0.5% Triton X-100. The cells were then blocked with 10% goat serum (Sangon Biotech, E510009) and subsequently incubated with primary antibodies and fluorescently labeled secondary antibodies. Green fluorescent puncta were observed under a fluorescence microscope.

### Western blotting

The myocardial tissues and cardiomyocytes were harvested from each mouse. After lysis, centrifugation, addition of sample buffer and boiling, protein samples were obtained. Following electrophoresis, the samples were transferred onto a membrane, sealed, and separately incubated with primary antibodies against tuberous sclerosis complex 1 (TSC1), phosphorylated mTOR (p-mTOR), total mTOR (t-mTOR), autophagy-related protein 3 (ATG3), ATG5, ATG7, Beclin-1, autophagy-related light chain 3 II (LC3II), lysosome-associated membrane glycoprotein (LAMP2), LAMP1, ATG14, ATG8, p62, cathepsin L, ATG12 and GAPDH at 4ºC overnight. Afterwards, horse-radish peroxidase (HRP)-labeled secondary antibodies (1:1,000) were added for incubation at room temperature for 1 h. Finally, the protein bands were determined by Bio-Rad imaging system and Image Lab software was utilized for quantitative analysis.

### Detection of autophagic flux via Western blotting

Cardiomyocytes in the logarithmic growth phase were seeded in 6-well plates at a density of 1.5 × 10^5^ cells/well, and the experimental groups were as follows: LPS group and LPS + miR-926-3p KD group. Cells were collected at different time points (6, 12, and 24 h) for Western blotting assay, as shown in Section 2.10.

### Statistical analysis

R v3.6.1 software package DEseq2 and statistical software package ggpubr were adopted for bioinformatics analysis. Wald test was employed for DEG analysis, and rank-sum test was utilized for comparison of cytokines between two groups. By SPSS 22.0 software, one-way analysis of variance was carried out to analyze the difference of each parameter among groups, and LSD-t test was used for pairwise comparison. Experimental data were expressed by mean ± standard deviation $(\bar{\text{χ}}\;\pm \;\text{s}).$* p* < 0.05 represented statistically significant differences.

## RESULTS

### Screening of DEGs

**Figure 1:**
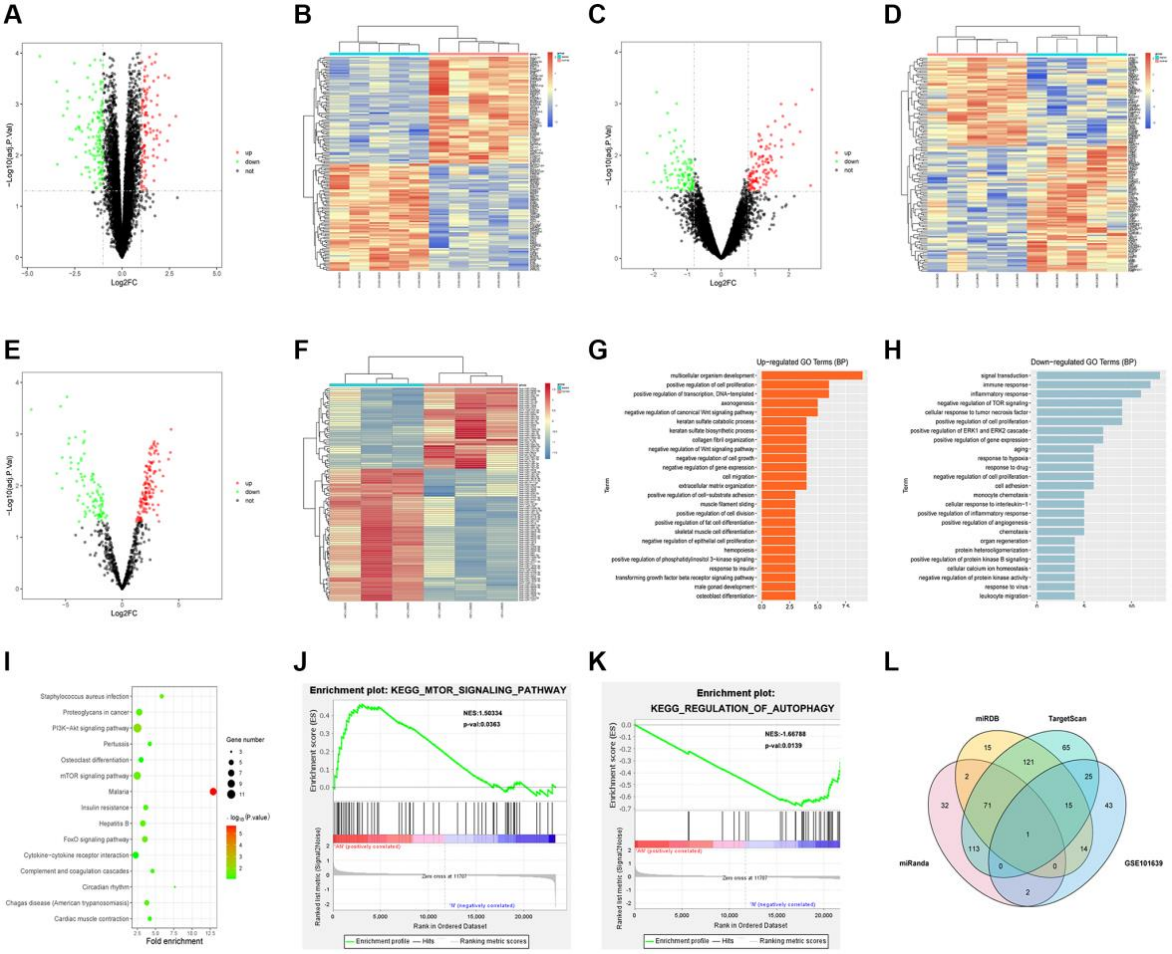
**Bioinformatics analysis.** (**A**) Volcano plot of visually grouped DEGs in GSE79962. (**B**) Cluster heatmap of DEGs in GSE79962. (**C**) Volcano plot of visually grouped DEGs in GSE12624. (**D**) Cluster heatmap of DEGs in GSE12624. (**E**) Volcano plot of visually grouped DEGs in GSE101639. (**F**) Cluster heatmap of DEGs in GSE101639. (**G** and **H**) Diagrams of up-regulated and down-regulated GO pathways of DEGs. (**I**) KEGG pathway of DEGs. (**J** and **K**) GSEA showed that regulation of autophagy, mTOR signaling pathway and other pathways were significantly related to the enrichment of DEGs. (**L**) Venn diagrams of candidate target genes and GSE101639 DEGs.

The sepsis-related datasets GSE79962 and GSE12624 and the miRNA dataset GSE101639 were downloaded from the GEO database. After screening of GSE79962 according to the criteria of *p* < 0.05 and |logFC|>1, 255 DEGs were found in sepsis mRNAs, including 123 up-regulated DEGs and 142 down-regulated DEGs. Then, the volcano plot of visually grouped DEGs in GSE79962 was drawn using ggplot2 software package in R software (Figure 1A), and the pheatmap software package of R was adopted to draw the cluster heatmap of DEGs (Figure 1B). As per the criteria of *p* < 0.05 and of DEGs (Figure 1B). As per the criteria of *p* < 0.05 and |logFC| > 0.8, the volcano plot (Figure 1C) and cluster heatmap (Figure 1D) of visually grouped DEGs in GSE12624 were drawn. Similarly, the volcano plot (Figure 1E) and cluster heatmap (Figure 1F) of visually grouped DEGs in GSE101639 were drawn based on the criteria of *p* < 0.05 and |logFC| > 0.5.

### Bioinformatics analysis

GO and KEGG enrichment analyses were conducted for DEGs from the intersection between GSE79962 and GSE12624. Besides, DEGs corresponding to biological processes were analyzed using the online database tool DAVID (https://david.ncifcrf.gov/) to integrate GO terms, and the biological process network of DEGs was created. Subsequently, the diagrams of up-regulated (Figure 1G) and down-regulated (Figure 1H) GO pathways and KEGG pathway (Figure 1I) of DEGs were plotted with R language. As shown in GO pathway diagrams, both up-regulated pathways (including multicellular organism development, positive regulation of cell proliferation, positive regulation of transcription, and DNA-templated) and down-regulated pathways (signal transduction, inflammatory response, and negative regulation of TOR signaling) were enrichment pathways for sepsis. The KEGG pathway diagram showed that enrichment was mainly found in such pathways as mTOR signaling pathway and cytokine-cytokine receptor interaction. Additionally, it was discovered through GSEA that regulation of autophagy, mTOR signaling pathway and other pathways were significantly related to the enrichment of DEGs (Figure 1J, 1K).

### Prediction of miRNA target genes

Candidate target genes were predicted using TargetScan, miRDB and miRanda online tools. Besides, Venn diagrams of these target genes and GSE101639 DEGs were drawn to find out co-binding miRNAs (Figure 1L).

### Changes in cardiac function-related indicators in each group of mice

**Figure 2:**
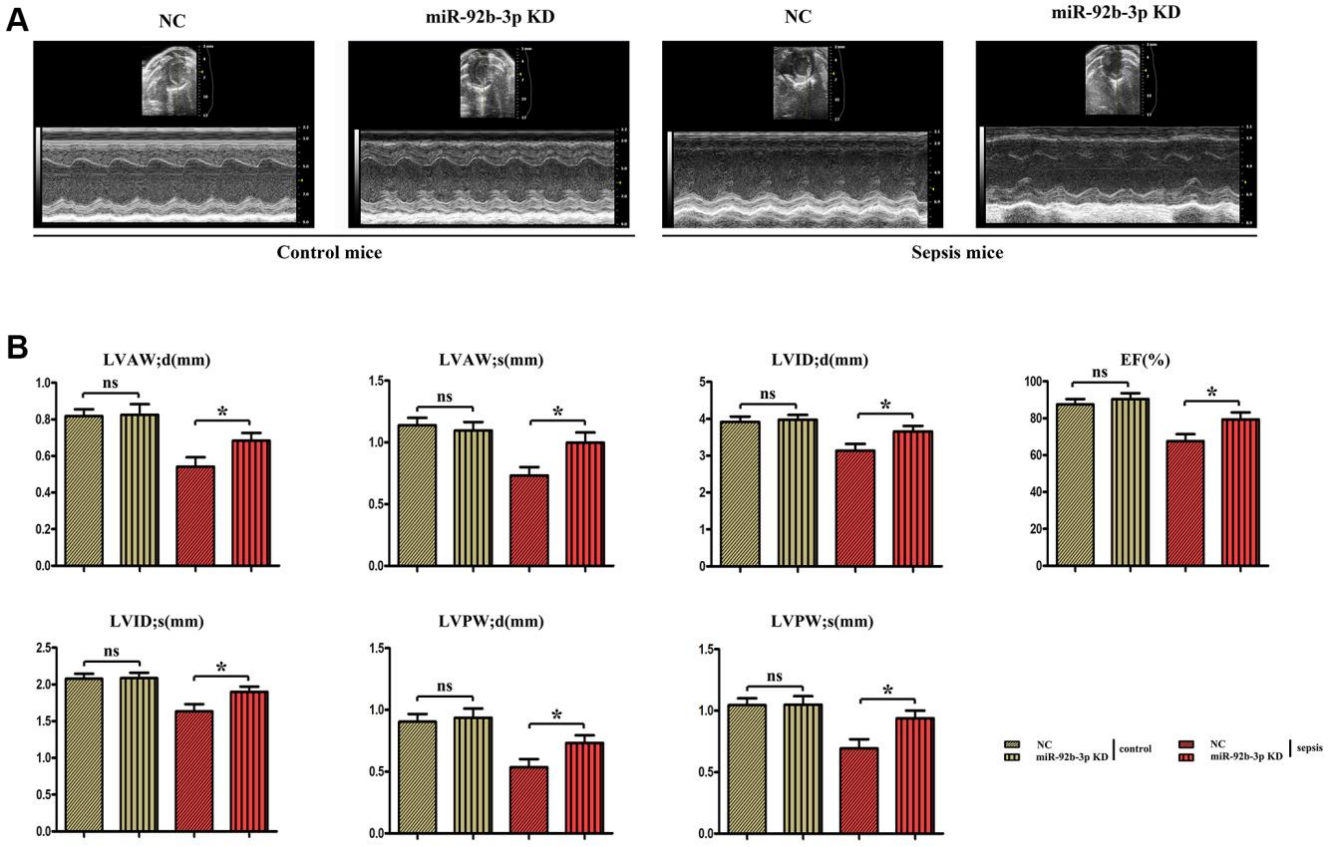
**The changes of cardiac function in mice were detected by ultrasound.** (**A**) Echocardiogram of the mouse heart. (**B**) Changes of echocardiographic parameters in mice. ^*^p < 0.05.

Ultrasound results uncovered that echocardiographic parameters had no evident changes in miR-926-3p inhibitor group compared with those in C group, while LVAW; d, LVAW; s, LVPW; d, LVPW; s, LVID; d, LVID; s and EF significantly declined in CLP + NC group. Besides, these indexes were obviously higher in CLP + miR-926-3p inhibitor group than those in CLP + NC group (Figure 2).

### Pathological changes of myocardial tissues in mice

**Figure 3:**
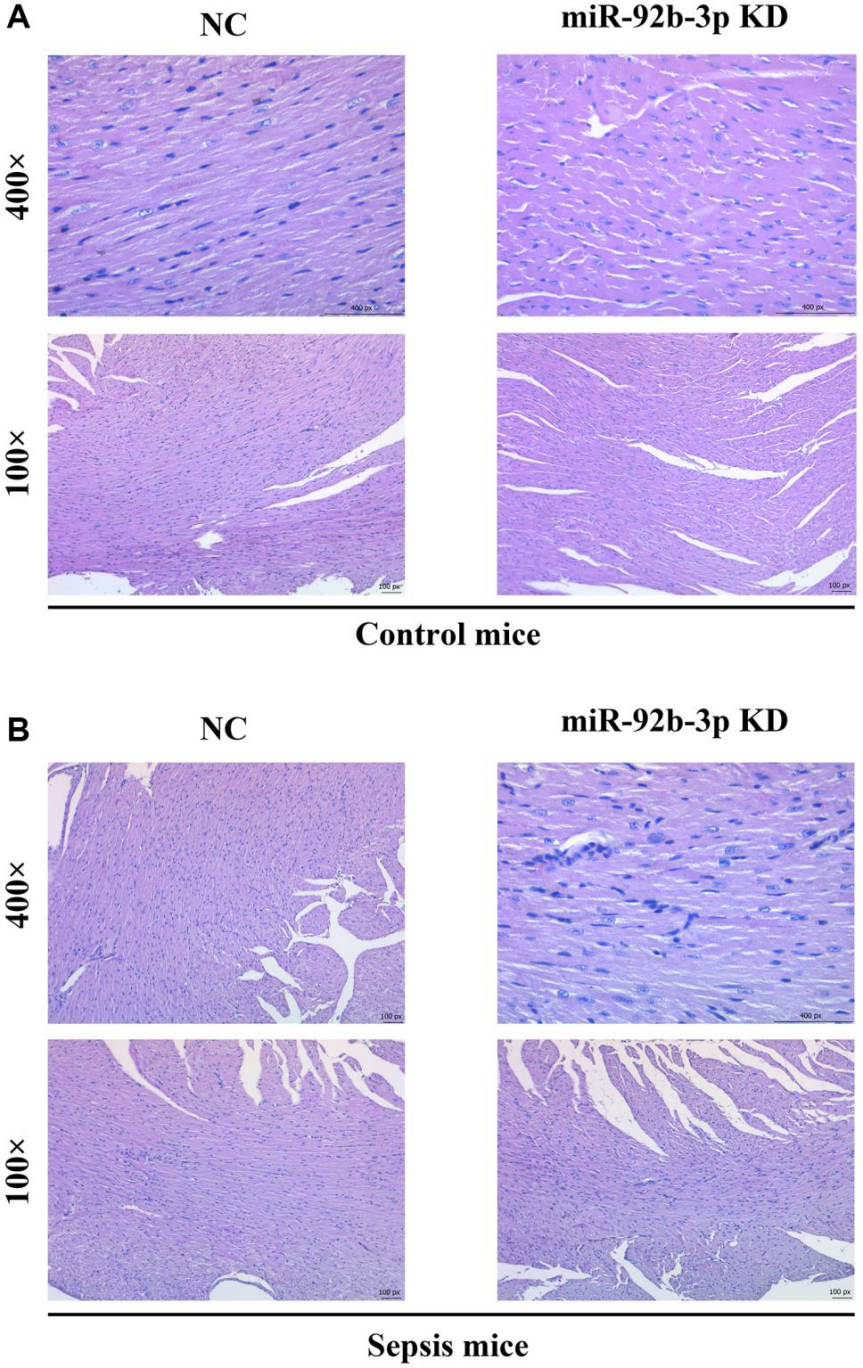
**Pathological changes of myocardial tissues in mice.** (**A**) Pathological results of central muscle tissue in mice in the control group; (**B**) Pathological results of mouse central muscle tissue in the sepsis group.

According to HE staining results, intact cardiomyocytes, clearly arranged muscle fibers and no infiltration of inflammatory cells were observed in C group and miR-926-3p inhibitor group. In CLP + NC group, blood vessels were dilated and congested, cardiomyocytes had focal degeneration and necrosis, and inflammatory cells infiltrated. In contrast with CLP + NC group, CLP + miR-926-3p inhibitor group exhibited improved pathological state of myocardial tissues, without obvious infiltration of inflammatory cells (Figure 3).

### Autophagy of myocardial tissues in mice observed by transmission electron microscopy

**Figure 4:**
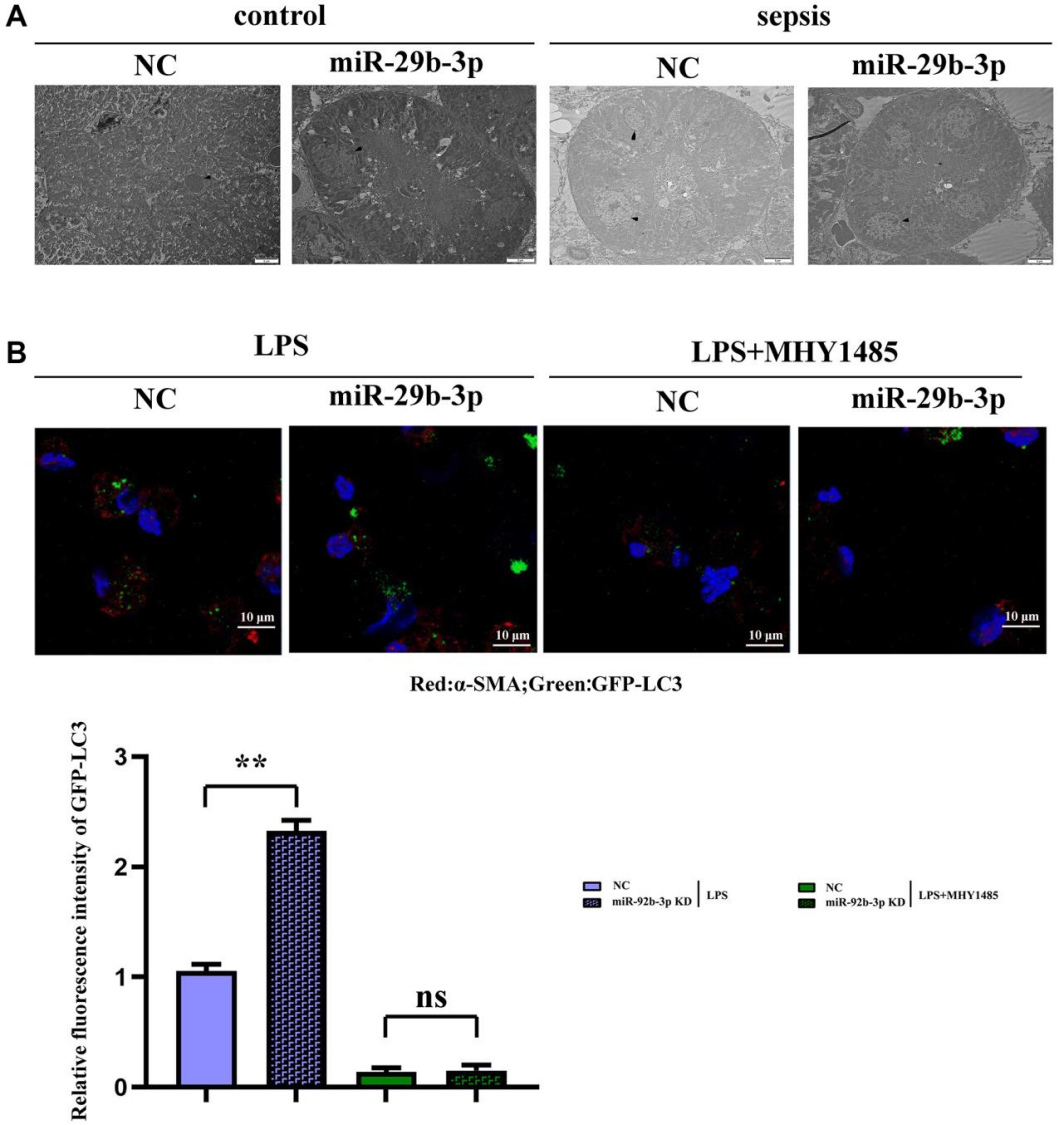
**Observation of myocardial autophagy in mice via electron microscopy and detection of GFP-LC3 puncta formation via fluorescence microscopy.** (**A**) Control group and the sepsis group used electron microscopy to observe the autophagy result plot; (**B**) Fluorescence plot of GFP-LC3 spots in each group and expression of relative fluorescence intensity. ^**^p < 0.01; ns *p* > 0.05.

It was observed by transmission electron microscopy that the autophagy phenomenon was weak and the number of lysosomes was small in C group and miR-926-3p inhibitor group, while CLP + NC group had autophagy enhancement relative to these two groups. Compared with those in CLP + NC group, autophagy enhancement and lysosome number increased in CLP + miR-926-3p inhibitor group, indicating that inhibition of miR-926-3p expression in sepsis can enhance autophagy (Figure 4A).

### Detection of GFP-LC3 puncta formation via fluorescence microscopy

Fluorescence microscopy revealed few green fluorescent puncta in C group and miR-926-3p inhibitor group. The number of green fluorescent puncta in CLP + NC group was higher than that in these two groups, while it was greatly increased in CLP + miR-926-3p inhibitor group as compared to CLP + NC group (Figure 4B).

### Low expression of miR-926-3p enhanced autophagy by regulating mTOR signaling pathway

**Figure 5:**
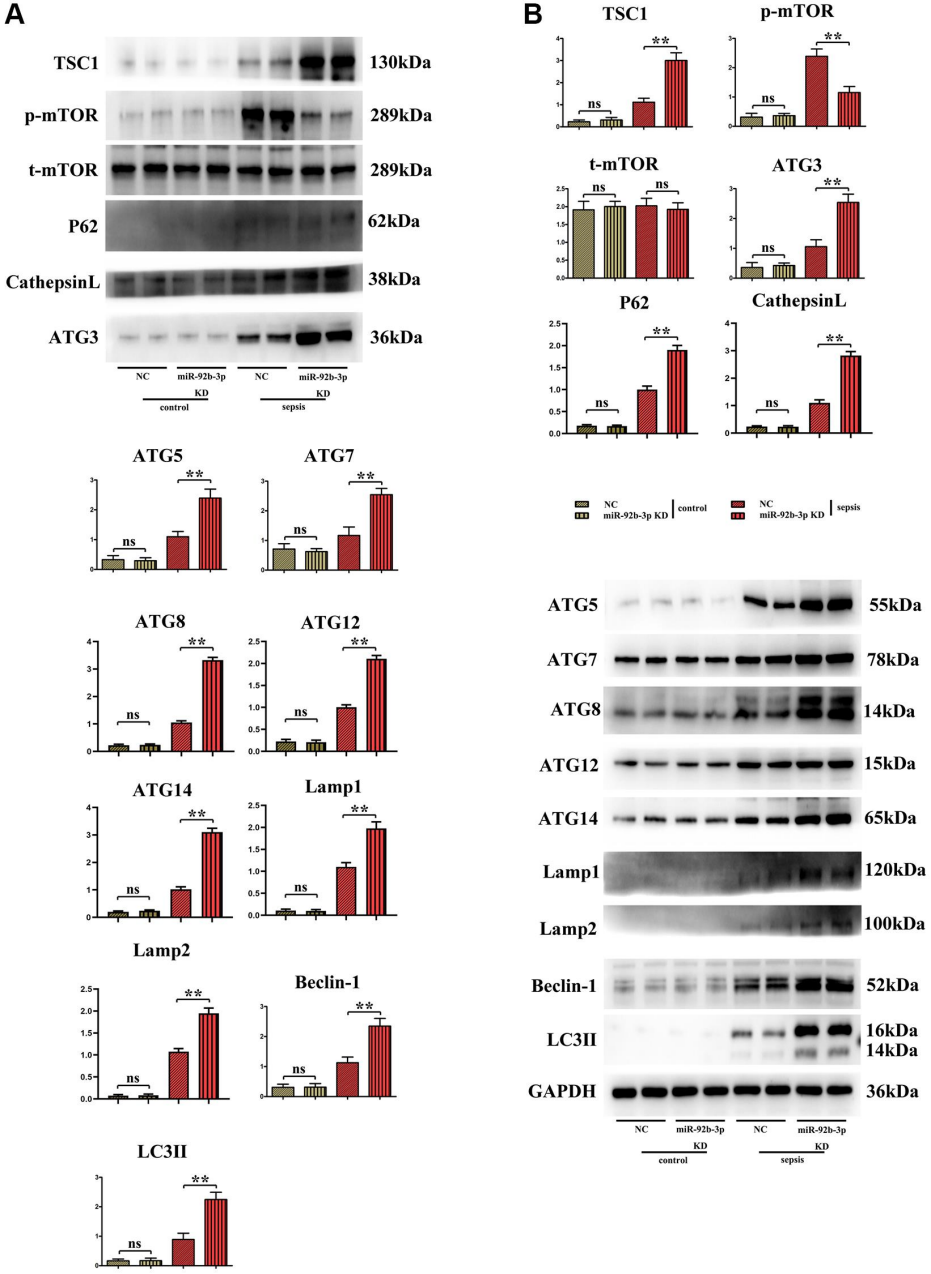
**Expressions of autophagy/mTOR pathway-related proteins in mouse myocardium.** (**A**) Relative expression of autophagy/mTOR pathway-related proteins in mouse myocardium; (**B**) Strip plot of autophagy/mTOR pathway-related proteins in mouse myocardium. ^**^p < 0.01; ns *p* > 0.05.

**Figure 6:**
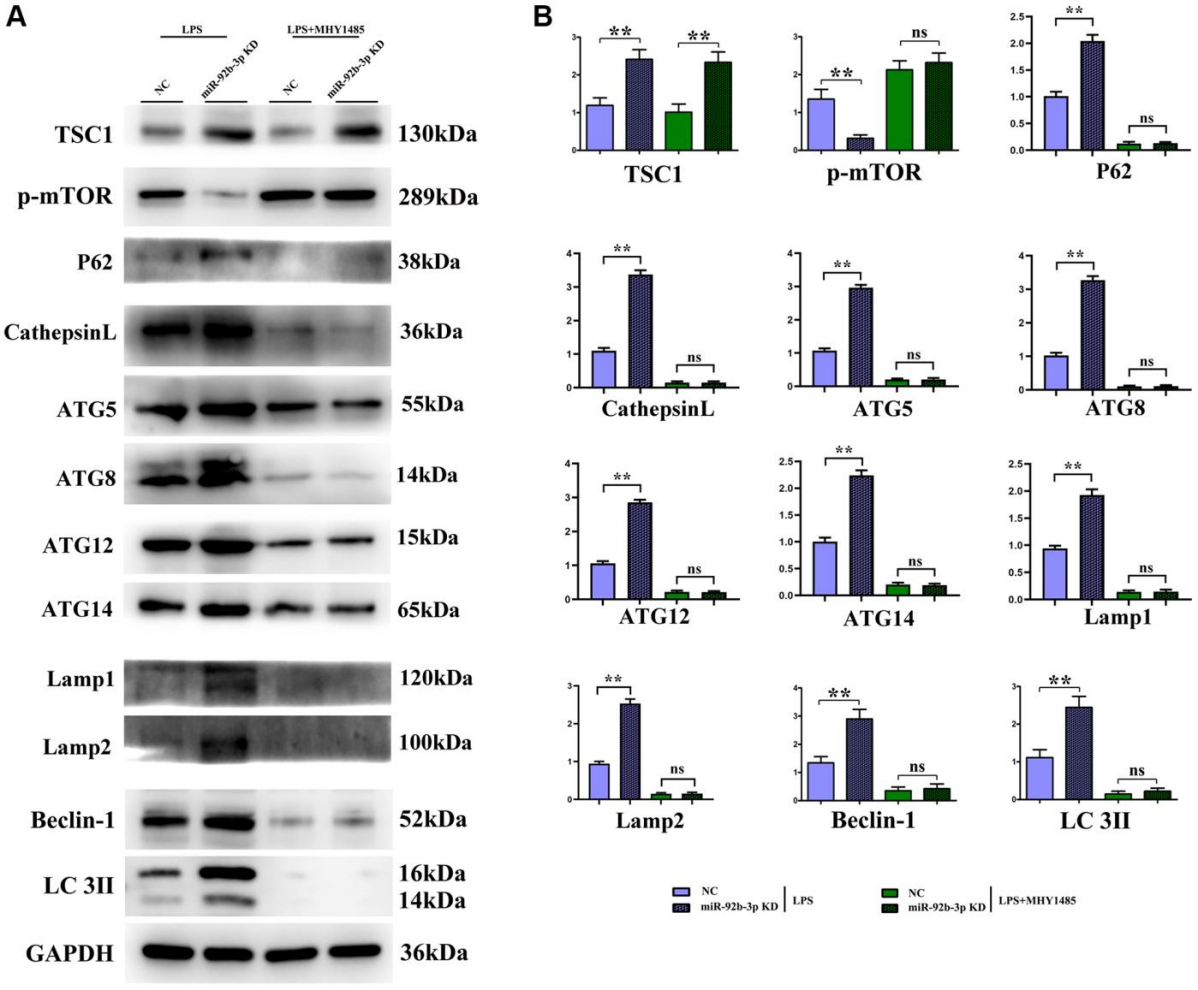
***In vitro* experiments demonstrated that low expression of miR-926-3p could enhance autophagy by regulating the mTOR signaling pathway.** (**A**) Strip plot for detection of mTOR pathway-related proteins in *in vitro* experiments. (**B**) *In vitro* experiments to detect the relative protein expression of proteins associated with the mTOR pathway. ^**^p < 0.01; ns *p* > 0.05.

It was found in Western blotting that in comparison with C group and miR-926-3p inhibitor group, mice receiving CLP displayed elevated protein expressions of TSC1, p-mTOR, ATG3, ATG5, ATG7, Beclin-1 and LC3II in myocardial tissues and no obvious changes in the protein expression of t-mTOR. In contrast with CLP + NC group, CLP + miR-926-3p inhibitor group had significantly increased protein expressions of TSC1, ATG3, ATG5, ATG7, Beclin-1, LC3II, LAMP1, LAMP2, ATG14, ATG12, ATG8, p62, cathepsin L and t-mTOR and a significantly reduced protein expression of p-mTOR in mouse myocardial tissues. In addition, the specific mechanism by which miR-926-3p expression affects the autophagy of cardiomyocytes in septic mice was investigated through LPS-induced cardiomyocyte injury *in vitro*. The results revealed that miR-926-3p inhibitor up-regulated the protein expressions of TSC1, Beclin-1 and LC3II and down-regulated p-mTOR expression in cardiomyocytes, consistent with the results of *in vivo* experiments. After adding MHY1485 (an mTOR agonist), the protein expression of p-mTOR in cardiomyocytes rose significantly in each group, while the expressions of autophagy-related proteins Beclin-1 and LC3II declined. Meanwhile, the protein expressions of p-mTOR, Beclin-1 and LC3II showed no significant differences between NC + MHY1485 group and miR-926-3p inhibitor NC + MHY1485 group. These results indicated that low expression of miR-926-3p regulates the mTOR signaling pathway to enhance autophagy (Figures 5, 6).

### Conversion of autophagy-related protein LC3II

**Figure 7:**
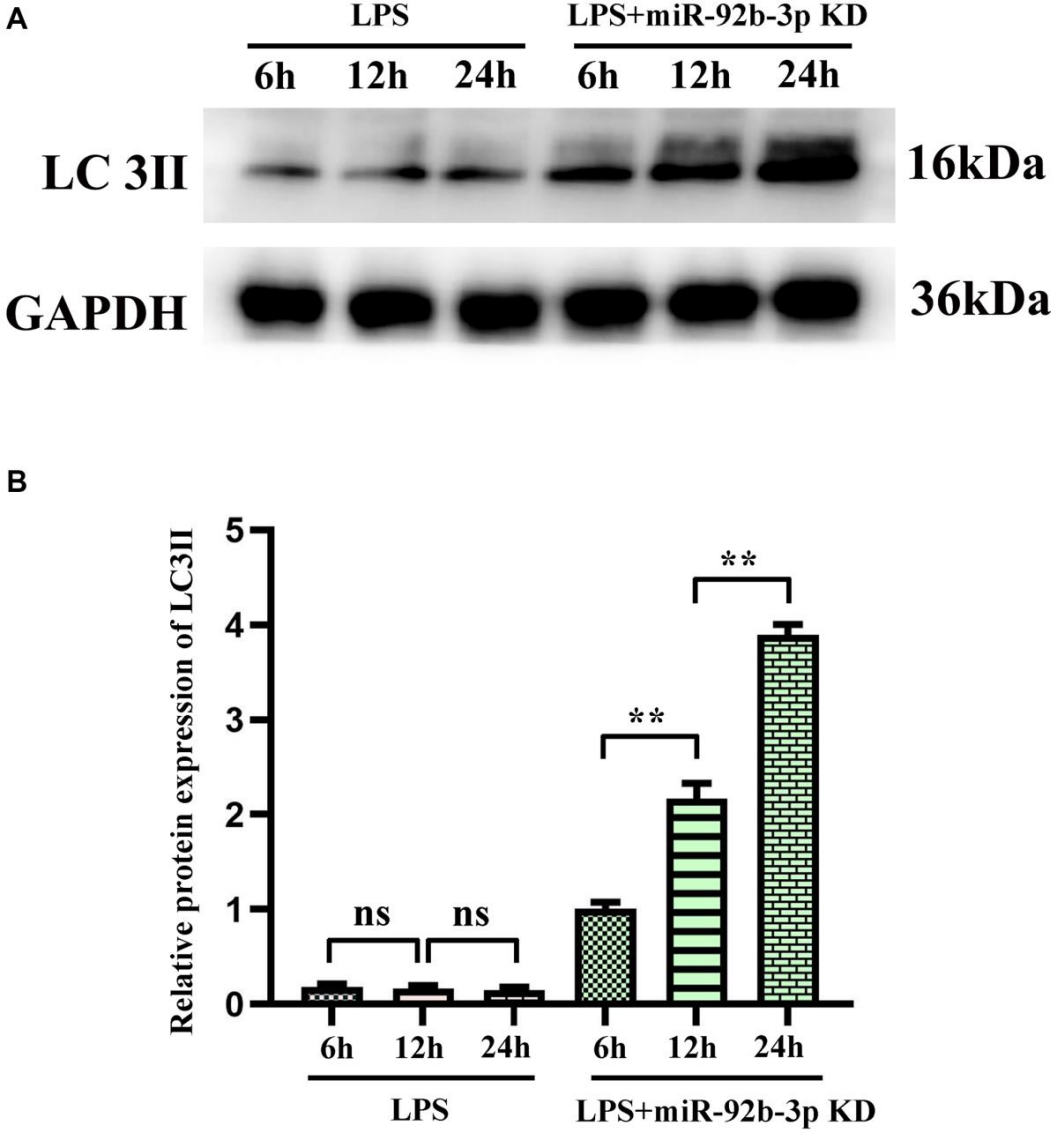
**Autophagy blockade of miR-926-3p KD on cardiomyocytes detected by Western blotting.** (**A**) Expression levels of autophagy-related protein LC3II in cardiomyocytes at different times. (**B**) Relative protein expression of LC3II. ^*^p < 0.05, ^**^p < 0.01.

The results of Western blotting assay showed that the protein expression of LC3II in cardiomyocytes was increased compared with that in blank control group at different time points after LPS treatment for 6, 12 and 24 h. The relative protein expression of LC3II was calculated and the ratio showed an upward trend with action time. Compared with that in LPS group, the protein expression of LC3II was increased in LPS + miR-926-3p KD group at the same time point. The above results manifested that after miR-926-3p KD blocks the degradation of autophagy at the lysosomal level, a significant increase in LC3II can be observed at the same time point compared with before blockade, that is, the autophagic flux increases (Figure 7).

## DISCUSSION

In this study, the mouse model of sepsis was induced through CLP, which is closer to the pathological process of patients in clinical practice and is a suitable model to explore the mechanisms of development and progression of sepsis [17]. Sepsis is a disease syndrome caused by pathogenic microorganism infection, eventually resulting in multiple organ failure. Severe sepsis and septic shock are major medical problems in treating critical patients [18, 19]. Sepsis mainly affects the heart and leads to serious adverse outcomes, and the prognosis of patients depends on the severity of myocardial injury [20]. Sepsis-induced myocardial injury, manifested by different types of cardiac dysfunction, causes a reduction in EF and an insufficient cardiac output, significantly increasing the risk of death [21]. In this study, it was also found that myocardial injury was obvious in mice with CLP-induced sepsis, with such manifestations as vascular dilation and congestion, focal degeneration and necrosis of cardiomyocytes, infiltration of inflammatory cells, and obvious apoptosis of cardiomyocytes (Figure 3). In addition, the cardiac function of septic mice was also severely impaired, with significantly decreased LVAW; d, LVAW; s, LVPW; d, LVPW; s, LVID; d, LVID; s and EF (Figure 2). After low expression of miR-926-3p, improved pathological changes in myocardial tissues and reversed echocardiographic parameters were observed in septic mice, suggesting that miR-926-3p inhibitor is able to improve LVAW; d, LVAW; s and LVPW; d of mice with myocardial injury, lowering the risk of pericardial tamponade due to ventricular wall rupture and hemorrhage. Moreover, the thinning of the ventricular wall may affect the left ventricular EF (%). EF as a major indicator for evaluating left ventricular systolic function can reflect the shortening of left ventricular myocardial fibers [17]. A significantly increased EF means that miR-926-3p inhibitor can alleviate cardiac dysfunction in septic mice. These results demonstrate that low expression of miR-926-3p protects against myocardial injury in septic mice.

In this study, therefore, the specific influencing mechanism of miR-926-3p on sepsis-induced myocardial injury in mice was further explored. A study manifested that sepsis can cause autophagy in multiple organs, including the heart [11]. The regulation of autophagy is a novel direction for the treatment of sepsis [15], in which the mTOR signaling pathway, a typical upstream node repressing autophagy, is a crucial player [22]. Combined with the results of bioinformatics analysis, miR-926-3p affected the activation of the mTOR pathway by targeting TSC1 (Figure 1). TSC, a heterodimer composed of TSC1 (also known as hamartin) and TCS2 (also known as tuberin), is capable of negatively regulating mTOR signal transduction through the small GTPase-activating protein (GAP) domain of TSC2 [23]. Figure 4 results showed that transmission electron microscopy observed that autophagy was weaker and the number of lysosomes was lower in the C group and miR-926-3p inhibitor groups, while the CLP+NC group was enhanced relative to these two groups. Compared with the CLP+NC group, the CLP+miR-926-3p inhibitor group was enhanced with enhanced autophagy and an increase in the number of lysosomes; Fluorescence microscopy showed that there were no green fluorescent spots in both group C and miR-926-3p inhibitor groups. The number of green fluorescent spots in the CLP+NC group was higher than in these two groups, while the number of green fluorescent spots in the CLP+miR-926-3p inhibitor group was significantly higher than in the CLP+NC group. Figure 7 showed that the relative protein expression of LC3II showed an increasing trend with the time of action. LC3II protein expression increased in the LPS+miR-926-3p KD group at the same time point compared to the LPS group. Figure 5 illustrated that compared with those in C group and miR-926-3p inhibitor group, the protein expressions of TSC1, ATG3, ATG5, ATG7, Beclin-1, LC3II, LAMP1, LAMP2, ATG14, ATG12, ATG8, p62, cathepsin L and p-mTOR in myocardial tissues were obviously elevated, while the protein expression of t-mTOR had no significant change in mice undergoing CLP. In contrast with CLP + NC group, CLP + miR-926-3p inhibitor group had significantly higher protein expressions of TSC1, ATG3, ATG5, ATG7, Beclin-1, LC3II, LAMP1, LAMP2, ATG14, ATG12, ATG8, p62, cathepsin L and t-mTOR and a significantly lower protein expression of p-mTOR in mouse myocardial tissues. Moreover, the specific mechanism by which miR-926-3p expression affects the autophagy of cardiomyocytes in septic mice was further investigated through LPS-induced cardiomyocyte injury *in vitro*, as shown in Figure 6. The results revealed that miR-926-3p inhibitor up-regulated the protein expressions of TSC1, Beclin-1 and LC3II and down-regulated p-mTOR expression in cardiomyocytes, consistent with the results of *in vivo* experiments. After adding the mTOR agonist MHY1485, the protein expression of p-mTOR in cardiomyocytes rose significantly in each group, while the expressions of autophagy-related proteins Beclin-1 and LC3II declined. These results suggest that the mTOR signaling pathway is involved in the regulation of autophagy in myocardial tissues of septic mice by miR-926-3p inhibitor. In brief, after miR-926-3p is lowly expressed, the expression of target gene TSC1 significantly rises, suppressing the activation of the mTOR signaling pathway, enhancing autophagy in myocardial tissues of septic mice, and attenuating myocardial injury in mice, which offers new ideas for therapies for sepsis.
