# Correction for: miR-926-3p influences myocardial injury in septic mice through regulation of mTOR signaling pathway by targeting TSC1

**DOI:** 10.18632/aging.206127

**Published:** 2024-09-30

**Authors:** Feng Yan, Qian Wang, Huiyu Yang, Hui Lv, Weiwei Qin

**Affiliations:** 1 Department of Cardiology, The Second Hospital of Shanxi Medical University, Shanxi 030001, People’s Republic of China

**Keywords:** miR-926-3p, TSC1, mTOR signaling pathway, sepsis, myocardial injury

**This article has been corrected:** The authors recently found that there is an error in the name of a microRNA described in the article. In the article, the microRNA was mistakenly referred as “miR-926-3p,” whereas the correct designation is “miR-92b-3p.” This error occurred in the title, text and figure legends, but not in the figures, where the microRNA was labeled “miR-92b-3p.” The corrected version of only the title and Abstract are provided below, but also in the text this microRNA should be read as “miR-92b-3p.” The authors would like to apologize for any inconvenience caused.

**miR-92b-3p influences myocardial injury in septic mice through regulation of mTOR signaling pathway by targeting TSC1**

**ABSTRACT**

Background: The purpose of this study is to investigate the influence of miR-92b-3p on myocardial injury and its mechanisms.

Methods: An animal model of sepsis was constructed by CLP, and animals were randomly divided into 4 groups: C group, miR-92b-3p inhibitor group, CLP + NC group, and CLP + miR-92b-3p inhibitor group.

Results: Compared with those in C group, echocardiographic parameters remarkably declined in CLP + NC group. Compared with CLP + NC group, miR-92b-3p inhibitor group indicated elevated echocardiographic parameters in mice, pathological improvement tendency of myocardial tissues and distinct reduction in cardiomyocyte apoptosis. It could be observed by electron microscopy that the number of lysosomes in miR-92b-3p inhibitor group was greatly increased relative to CLP + NC group. Immunofluorescence exhibited that the number of green fluorescent puncta was significantly higher in miR-92b-3p inhibitor group as compared to that in CLP + NC group. The autophagic flow was verified by observing the relative expression of LC3II at different times. The results of Western blotting manifested that miR-92b-3p inhibitor up-regulated mTOR-related protein expressions and down-regulated the protein expression of p-mTOR. LPS was adopted to induce cardiomyocyte injury *in vitro*, and the results confirmed that, identical to *in vivo* experiments, miR-92b-3p inhibitor was able to up-regulate the protein expressions of mTOR-related protein and down-regulate p-mTOR protein expression in cardiomyocytes. After addition of MHY1485, The expression of mTOR-related proteins changes in each group.

Conclusion: Inhibition of miR-92b-3p enhances autophagy through regulation of the mTOR signaling pathway, thus ameliorating myocardial injury in septic mice.

